# Opioid-Free Anesthesia for Open Radical Cystectomy Is Feasible and Accelerates Return of Bowel Function: A Matched Cohort Study

**DOI:** 10.3390/jcm12113657

**Published:** 2023-05-25

**Authors:** John-Patrik Burkhard, François Jardot, Marc A. Furrer, Dominique Engel, Christian Beilstein, Patrick Y. Wuethrich

**Affiliations:** 1 Department of Anesthesiology and Pain Medicine, Inselspital, University Hospital Bern, University of Bern, 3010 Bern, Switzerland; 2 Limmat Cleft- and Craniofacial Centre Zurich, 8005 Zurich, Switzerland; 3 Department of Urology, Solothurner Spitäler AG, Kantonsspital Olten, Bürgerspital Solothurn, 4500 Solothurn, Switzerland; 4 Department of Urology, Inselspital, University Hospital Bern, University of Bern, 3010 Bern, Switzerland

**Keywords:** opioid-free anesthesia, multimodal anesthesia, gastrointestinal function, opioid consumption

## Abstract

The aim of this study was to evaluate the feasibility of opioid-free anesthesia (OFA) in open radical cystectomy (ORC) with urinary diversion and to assess the impact on recovery of gastrointestinal function. We hypothesized that OFA would lead to earlier recovery of bowel function. A total of 44 patients who underwent standardized ORC were divided into two groups (OFA group vs. control group). In both groups, patients received epidural analgesia (OFA group: bupivacaine 0.25%, control group: bupivacaine 0.1%, fentanyl 2 mcg/mL, and epinephrine 2 mcg/mL). The primary endpoint was time to first defecation. Secondary endpoints were incidence of postoperative ileus (POI) and incidence of postoperative nausea and vomiting (PONV). The median time to first defecation was 62.5 h [45.8–80.8] in the OFA group and 118.5 h [82.6–142.3] (*p* < 0.001) in the control group. With regard to POI (OFA group: 1/22 patients (4.5%); control group: 2/22 (9.1%)) and PONV (OFA group: 5/22 patients (22.7%); control group: 10/22 (45.5%)), trends but no significant results were found (*p* = 0.99 and *p* = 0.203, respectively). OFA appears to be feasible in ORC and to improve postoperative functional gastrointestinal recovery by halving the time to first defecation compared with standard fentanyl-based intraoperative anesthesia.

## 1. Introduction

Open radical cystectomy (ORC) with extended pelvic lymph node dissection and urinary diversion remains the standard of care for patients with muscle-invasive bladder cancer or recurring high-risk non-muscle-invasive bladder cancer [1]. This is considered as a major abdominal procedure associated with a high incidence of postoperative complications of approximately 60%, most commonly affecting the gastrointestinal system in up to 25% of all cases [2]. Enhanced recovery after surgery (ERAS) protocols are comprehensive perioperative care plans that aim to promote swift recovery post-surgery by mitigating pain and nausea, reducing wound infections and hospital stays, and expediting the restoration of bowel function [3,4].

Accordingly, perioperative thoracic epidural anesthesia (TEA) remains a key component in the matter of improving clinically oriented outcomes [5,6]. This is not only because of its excellent analgesic properties [7], but also due to the reduction in the postoperative stress response, faster return of bowel function [8], and lower postoperative morbidity and mortality [9,10,11,12]. Nevertheless, the use of TEA is a matter of discussion for ORC as the benefit is debatable [13]. TEA is readily used in major abdominal surgical procedures in combination with general anesthesia. The main advantage is the fact that the administration of systemic opioids and other analgesics can be significantly reduced [14]. 

Opioids are still widely used for both analgesia and supplementary sedation during general anesthesia. Furthermore, they are the most commonly used agents for the treatment of acute pain in the immediate postoperative period. Concerns about unnecessary use of opioids are excessive perioperative opioid consumption, potential narcotic dependence, respiratory depression, nausea and vomiting, and postoperative ileus (POI). These complications are associated with delayed patient recovery and prolonged length of stay, resulting in an increased burden on patients and health resources. 

Because of the known adverse effects of opioids in the perioperative period, their use should be kept to a minimum. The Enhanced Recovery after Surgery (ERAS) Society is also working on strategies to decrease the frequency of opioid administration.

This gave rise to the concept of opioid-free anesthesia (OFA), in which either multimodal non-opioid analgesic techniques or regional anesthetic techniques are implemented to avoid the intraoperative administration of opioids. The feasibility of OFA has been described in various major surgeries, but its feasibility and possible benefits in cystectomy patients remain largely unexplored [15,16,17,18]. 

The purpose of this study was to evaluate the feasibility of OFA in ORC and to assess the impact on recovery of bowel function assessed by time to first flatus and first defecation, as well as incidence of POI and postoperative nausea and vomiting (PONV).

## 2. Materials and Methods

This retrospective observational matched case series study reports a consecutive case series from a single tertiary center and is in accordance with the Strengthening the Reporting of Observational Studies in Epidemiology (STROBE) statement. Ethical approval for this study was provided by the Ethical Committee of the Canton Bern, Switzerland (KEKBE 2016-00660) on 2 June 2016, and informed consent was waived.

Patients included as comparators were enrolled in this study, which was approved by the local ethics committee (151/13) and by the Swiss Agency for Therapeutic Products (2014DR4097) [19], prospectively registered at http://www.controlled-trials.com, (accessed on 14 August 2020), (ISRCTN32976792), and conducted in accordance with the Declaration of Helsinki and Good Clinical Practice. All patients gave prior written informed consent.

### 2.1. Study Population

We identified 71 consecutive patients who underwent ORC and urinary diversion at the Department of Urology University Hospital Bern between 1 January 2019 and 31 December 2019, 22 of whom received OFA and were compared to a historical series of 22 patients. The indication for OFA was at the discretion of the attending anesthesiologist, and there was no contraindication for the drugs administered (ketamine, dexmedetomidine (sino-, atrio-, or intra-ventricular block; β-blocker treatment and heart rate < 50 beats·min^−1^; and cardiac insufficiency), and epidural analgesia (refusal of the patient and coagulation disorder)). All patient data were evaluated from a prospectively maintained cystectomy database that fully complies with the legal requirements of the Federal Human Research Act. Patients and procedures from 1 January 2019 to 31 December 2019 were manually extracted from the patient database and retrospectively completed from the patients’ paper charts and anesthetic protocols. 

Patients of the comparison group were screened for eligibility and recruited from July 2014 to May 2015 for a randomized, parallel, single-center trial. From this trial, only patients of the control group were included as comparators because they received the same standard intraoperative fluid as the control group: a balanced Ringer’s maleate solution (Ringerfundin^®^, B. Braun, Sempach, Switzerland) [19].

In addition, outcomes of this randomized clinical trial (RCT) were focused on the return of gastrointestinal function, as well as flatus, first defecation, and incidence of POI, so these were exactly assessed.

### 2.2. Surgical Technique and Perioperative Management

No antegrade bowel preparation or enemas were administered preoperatively. Patients had oral food intake until midnight before surgery and were urged to drink clear liquids, including carbohydrated oral fluids, until 2 h before induction of anesthesia.

At our institution, ORC, extended pelvic lymph node dissection, and urinary diversion (ileal orthotopic bladder substitutes, an ileal conduit, and a continent catheterizable ileal reservoir) have been performed for the last 10 years using the same standardized surgical technique, as previously described, and all patients were followed prospectively [20,21].

After cystectomy, a distal ileal segment was isolated via a standard intraperitoneal approach and urinary diversion was performed. A gastrostomy tube was placed intraoperatively, and the orogastric tube was removed at the end of the procedure.

A protocol for systematic restrictive fluid administration was followed, with a fluid maintenance (a lactated or maleated Ringer’s solution) of around 1 to 3 mL per kg body weight per hour (mL·kg^−1^·h^−1^) of crystalloids. If necessary, a low-dose continuous administration of norepinephrine was started at approximately 1 to 2 µg·kg^−1^·h^−1^ to achieve a mean arterial blood pressure between 60 mmHg and 100 mmHg. Blood loss was primarily replaced with crystalloids. Packed red blood cells were transfused when hemoglobin levels decreased perioperatively to <80 gL^−1^ (<100 gL^−1^ in patients with coronary artery disease). The perioperative administration of fresh frozen plasma was guided primarily by the observed bleeding in the surgical field and by agreement between the anesthesiologist and urologist in charge and secondly by the number of packed red blood cells administered. 

Postoperative hydration consisted of 1000 mL of a Ringer’s lactate solution and 500 mL of glucose 5% per day until resumption of normal food intake [22]. In case of hypotension, first-line treatment was an additional bolus of 250–500 mL of the lactated Ringer’s solution. Patients were allowed to drink clear fluids in the immediate postoperative period.

On the first postoperative day, the oral liquid diet was increased and active mobilization was started. The approach to support postoperative gastrointestinal function was standardized and in accordance with our internal ERAS guidelines for cystectomy patients, including the recommendation of chewing gum use [23]. The gastrostomy tube was initially left on the drain, and the gastrostomy tube was removed when there was no nausea or vomiting for more than 24 h. Esomeprazole was administered for 48 h. Oral fluid intake included energy drinks on the first day. As part of the protocol, subcutaneous neostigmine was administered as a prokinetic at a dosage of 0.25 to 0.5 mg up to three times a day, alongside oral laxatives starting from day 2. The introduction and promotion of small snacks or purees was marked on day 2, but no later than day 3.

Bedside mobilization was encouraged as soon as possible, ideally the same evening after surgery; if this was not possible—at the latest the next morning. Active ambulation, including [15,16,17,18] exercising in bed, sitting out of bed, and standing and walking in the room was started on postoperative day (POD) 1, and prolonged mobilization as well as sitting in a chair were started on POD 2.

### 2.3. Anesthesia and Perioperative Analgesic Management

Standard intraoperative monitoring involved continuous electrocardiographic data, heart rate, nasopharyngeal core temperature, pulse oximetry, invasive mean arterial pressure with a radial artery catheter, and central venous pressure with a venous catheter inserted in the right internal jugular vein. 

#### 2.3.1. OFA Group

Anesthesia was induced with propofol (2 mg·kg^−1^), ketamine (0.3 mg·kg^−1^), and rocuronium (0.6 mg·kg^−1^), and maintained with sevoflurane at an age-corrected minimum alveolar concentration of 0.6 combined with a continuous administration of dexmedetomidine at a continuous maintenance rate of 0.3 to 0.5 mg·kg^−1^·h^−1^. The dexmedetomidine dosage was adjusted to heart rate and then reduced when the total dose was up to 1.5 mg·kg^−1^ ideal body weight. The dexmedetomidine infusion was stopped at the onset of closure of the peritoneum. No opioids in any form were administered intravenously or epidurally during the whole surgical procedure and in the postoperative period. An epidural catheter was placed at the T9/T10 level in all patients. An 18-gauge epidural needle was inserted and the epidural space was identified with the loss-of-resistance technique. After a test dose of 1.5 mL lidocaine 2% with 0.005 mg·mL^−1^ epinephrine to rule out a subarachnoid or intravascular placement, a 0.25% bupivacaine infusion was administrated at a rate of 6–8 mL·h^−1^. TEA remained until POD 5 to 7 combined with an opioid-free systemic analgesia (paracetamol and metamizole). For OFA patients, the epidural solution was changed during closure of the abdominal wall to bupivacaine 1.25% administered by a CADD Legacy ambulatory infusion pump (model 6300, Deltec Inc., St Paul, MN, USA). The initial infusion rate was 8 mL·h^−1^, with a maximum infusion rate of 15 mL·h^−1^, and with additional bolus doses of 5 mL (lockout time: 1 h) from the end of surgery until 8 h in the morning on POD 3. The infusion rate could be adjusted if needed to maintain a NRS of <3 at rest and <5 during mobilization based on four hourly assessments [24].

#### 2.3.2. Control Group

Anesthesia was induced with propofol (2 mg·kg^−1^), fentanyl (2 µg·kg^−1^), and rocuronium (0.6 mg·kg^−1^), and maintained with isoflurane at an age-corrected minimum alveolar concentration of 0.6 and boluses of fentanyl. Normothermia was maintained with a convective air warming system (Bair Hugger™, 3M™-Switzerland, Rüschlikon, Switzerland) and using a Hotline^®^ fluid warmer (Smith Medical International Ltd., Ashford, Kent, UK). The epidural catheter was placed with a similar technique to that for the patients in the OFA group and at the same level (T9/T10), including an identical test dose. A 0.25% bupivacaine infusion was administrated at a rate of 6–8 mL·h^−1^ intraoperatively. 

Postoperatively, the patients in the control group received our standard epidural solution containing bupivacaine 1%, fentanyl 2 mcg/mL, and epinephrine 2 mcg/mL using the same system. The infusion rate, maximum infusion rate, and additional bolus doses with lockout time were similar to those implemented for the patients in the OFA group. The infusion rate could be adjusted if needed to maintain a NRS of <3 at rest and <5 during mobilization based on four hourly assessments [24].

### 2.4. Assessment of Postoperative Return of Bowel Function

In order to examine the primary endpoint of the return of bowel function, time to first flatus and first defecation, as well as incidence of POI, were calculated. POI was defined as no return of bowel function after postoperative POD 6, requiring cessation of oral intake, intravenous support, or nasogastric tube placement [25]. Incidence of PONV and antiemetic use were recorded [26]. All patients with PONV episodes received antiemetics (intravenous ondansetron and droperidol). Complete follow-up data were available for all participants.

### 2.5. Statistical Analysis

Data showing a normal distribution are presented as the mean ± standard deviation (SD). Elsewhere, data are reported as medians with interquartile ranges [IQR] for continuous variables and frequencies for categorical variables. We performed exploratory landmark analyses for categorical data using the Fisher’s exact test or the chi-square test, and for continuous data using the Mann–Whitney U test.

A *p* < 0.05 was considered significant for all statistical tests. The statistical software used was SPSS version 28.0.0 for Mac (IBM Corp., Armonk, NY, USA). Based on our precedent data and the power calculation for the RCT by Loeffel et al. [19], we stated that a sample size of 18 patients per randomized arm would have a power of 90% (β = 0.10) to detect a difference of 1 day between the groups at a two-sided significance level of 5% (α = 0.05), assuming a SD of 1 day.

## 3. Results

Patients in the OFA group were significantly older (median age: 70.8 years [IQR: 63.1–77.1] vs. the control group, 64.0 years [56.5–70.5]). Perioperative risk stratification showed a markedly higher comorbidity, assessed with the American Society of Anesthesiologists physical (ASA) scores for physical status: in the OFA group, 73% were ASA III and IV compared to 50% in the control group (Table 1). Mean intraoperative administration of fentanyl in the control group was 483 µg (SD ± 256); wherein their overall fluid administration intraoperatively was similar (1950 mL [1325–2500]) compared with the OFA group (2100 [1487–2600]), *p* = 0.724. Baseline characteristics are presented in Table 1. 

**Figure 1:**
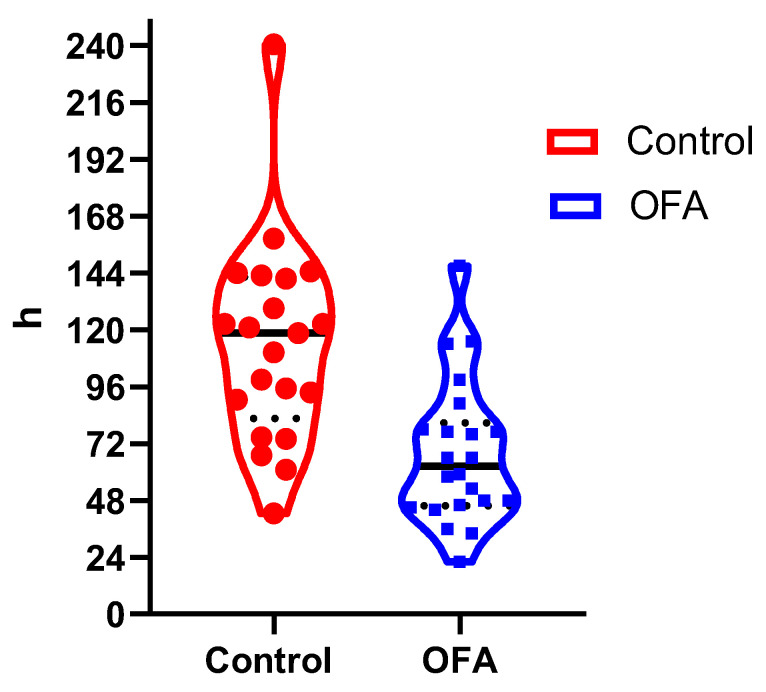
Violin plot of the time to first defecation, points in colors illustrate each patient, *p* < 0.0001.

The primary endpoint—the return of bowel function, as measured by the time to first defecation, differed by a factor of two. In the OFA group, the median time was 62.5 h [IQR: 45.8–80.8], which is significantly less than the 118.5 h [82.6–142.3] measured in the control group (Figure 1 and Table 2). 

Regarding secondary endpoints, in contrast, the occurrence of first flatus did not differ significantly (Table 2). POI occurred in 1 of 22 patients (4.5%) in the OFA group and in 2 of 22 patients in the control group (9.1%), *p* = 0.99. Fluid balance assessed on POD 2 and 3 was similar in both groups (Table 2). The incidence of at least one episode of PONV was 23% (5/22 patients) in the OFA group and 46% (10/22) in the control group, *p* = 0.203. 

No side effects related to the drugs administered in the OFA group (i.e., ketamine and dexmedetomidine) could be detected. In particular, neither a severe arrhythmia and/or bradycardia nor delayed extubation could be found.

## 4. Discussion

This case series demonstrates that TEA-based OFA is feasible for ORC patients and can significantly accelerate the return of bowel function (first defecation), potentially reducing the absolute risk of gastrointestinal complications. 

Delayed recovery of bowel function or dysfunction precisely is a relevant postoperative complication in patients undergoing ORC and urinary diversion, occurring in up to 25% of all cases [27,28]. This occurs despite perioperative strategies such as early oral nutrition, intravenous fluid administration, prokinetics, and restrictive opioid use for pain control [5,19,23,26,29,30,31,32]. In this context, perioperative use of TEA seems to positively influence the return of gastrointestinal function by reducing or completely avoiding the need to administer systemic opioid-containing medication during anesthesia [14]. This impression is supported by the present study, as our results suggest that OFA may shorten the time to first defecation. The underlying concept is based on the goal that OFA eliminates the negative effects of intraoperative opioids on the patient’s postoperative outcome and on the physiology of the neural pathways involved in intraoperative nociception [33]. It is based on the concept of multimodal anesthesia and has shown to be feasible in various invasive surgical procedures and disciplines [16,18]. However, the term OFA implies different strategies widely ranging from drug regimens with multimodal administration of systemic medications such as lidocaine, magnesium, ketamine, and beta-blockers up to the implementation of regional techniques. Even in terms of regional techniques, there is an array of different approaches including single shot blocks (spinal and peripheral) or those including implantation of catheters (epidural or continuous wound infiltrations) [16,17]. This variability in treatment regimens has led to the wide range of different outcomes. A well-designed prospective study demonstrating the benefit of OFA remains controversial. In a recent RCT, OFA significantly enhanced the quality of recovery after surgery (assessed with the QoR40 questionnaire) in patients undergoing laparoscopic gynecological surgeries [34]. Massoth et al., in the frame of a RCT involving 152 participants, concluded that OFA, although feasible for gynecologic laparoscopic surgery, did not reduce the incidence of PONV, pain scores, or morphine consumption compared with an opioid-containing anesthetic regimen [18]. Beloeil et al., who examined the hypothesis that balanced OFA with dexmedetomidine would result in fewer postoperative opioid-related adverse events compared with remifentanil, showed a higher incidence of serious adverse events, particularly hypoxemia and bradycardia [15]. Thus, the literature on the use of OFA is very heterogeneous in its results and difficult to compare, whereas studies on ORC are still lacking. A retrospective case series was able to illustrate a benefit in terms of fewer infectious complications in a French cohort; however, this study included patients undergoing minimally invasive robotic-assisted radical cystectomy [35]. 

One reason why the potential benefit of OFA cannot be clearly demonstrated is that it is a multimodal anesthesia; it uses a mixture of several drugs and this polypharmacy is not without concerns. The French multicenter RCT mentioned above, led by the NOFA study group, had to be stopped prematurely because of severe side effects due to the administration of dexmedetomidine [15]. In our series, we could not detect severe bradycardia, nor the administration of dexmedetomidine resulting in delayed extubation. 

Our OFA concept is based on the optimal use of epidural analgesia and anesthesia while avoiding the administration of opioids during induction. Polypharmacy was minimized by maintaining anesthesia with only sevoflurane and a low dose of dexmedetomidine. Continuous infusion of dexmedetomidine was also reduced when the total dose reached 1.2 mg·kg^−1^ ideal body weight and then stopped 30 min before peritoneal closure. In this way, concerns about potentially increased drug toxicity were reduced in a multimodal approach. We are aware that the use of TEA is becoming more critical as other less invasive alternatives are valuable options (local infiltration with liposomal bupivacaine, transversus abdominis plane (TAP) blocks, and wound catheters). One argument against the use of TEA is that patients cannot be mobilized because of hypotension or orthostatic disturbances. However, this can be reduced by epidural administration of a triple mixture composed of bupivacaine 0.1%, epinephrine 2 µg·mL^−1^, and fentanyl 2 µg·mL^−1^. Adding epinephrine to the mixture results in the relevant systemic concentration of all epidurally administered drugs. In addition, the mode of continuous administration could also be relevant. A programmed intermittent epidural bolus contributes to reducing the side effect of hypotension and leads to better analgesia, which has been demonstrated in obstetric patients (walking epidural) [36].

It is also unclear whether the administration of dexmedetomidine per se has a positive impact on the return of bowel function. Lu et al. showed, in a RCT including more than 800 patients undergoing major gastrointestinal surgery, that the onset timing of the first flatus and first defecation was significantly shorter in patients treated with dexmedetomidine. Subsequently, they used an almost similarly low dose maintenance rate of 0.2 μg·kg^−1^·h^−1^ of dexmedetomidine but with a loading dose of 0.5 μg·kg^−1^ over 15 min. The authors argue that the positive influence of dexmedetomidine was based on the low-dose maintenance rate of dexmedetomidine, acting on central α2-adrenergic receptors to reduce the sympathetic tone. This could have enhanced the beneficial impact of TEA as epidurally administered local anesthetics such as bupivacaine are known to induce a segmental sympathetic block [37]. 

Limitations of the current study are that this trial was not randomized, but was a facility-specific cohort design with a relatively limited number of patients. However, the post-hoc calculated sample size indicates that it is unlikely that the results can be associated with a sample size that is too small. Another limitation is the large interval between the observations in the two groups. Inclusion bias cannot be ruled out for patients in the OFA group. However, as these patients were older and had higher ASA scores, it is not obvious that only a positive selection of “healthy” patients was made.

A strength of this case series is that the primary outcome, the return of bowel function, was assessed by nurses who were not involved in either the original RCT with the control group or OFA group, as this assessment was part of daily nurses’ daily documentation. 

## 5. Conclusions

OFA combining TEA and a low-dose maintenance rate of dexmedetomidine appears to be feasible for an open major abdominopelvic surgery such as open radical cystectomy with urinary diversion, which is a kind of surgery associated with a high incidence of gastrointestinal complications. OFA has the ability to improve several meaningful parameters of postoperative functional gastrointestinal recovery shown by the early return of bowel function (first defecation).

## Figures and Tables

**Table 1:** Demographics and intraoperative parameters.

**Demographic Parameters**				
	**All**	**OFA (*n* = 22)**	**Control (*n* = 22)**	***p*-Value**
Age (years)	68.1 [59.2–75.3]	70.8 [63.1–77.1]	64.0 [56.5–70.5]	0.025
Female/Male	17/27	9/13	8/14	0.999
BMI (kg/m^2^)	25.6 [21.8–27.7]	24.1 [21.2–27.5]	25.9 [24.1–27.8]	0.215
ASA Physical Score II/III/IV	19/22/1	5/15/1	14/7/0	0.007
**Intraoperative Parameters**				
	**All**	**OFA (*n* = 22)**	**Control (*n* = 22)**	***p*-Value**
OP Time (min)	368 [337–423]	355 [325–422]	405 [343–450]	0.181
Blood Loss (mL)	810 [600–1500]	765 [500–1310]	1200 [700–1800]	0.055
Fluids (mL)	1100 [950–2100]	2100 [1487–2600]	1950 [1325–2500]	0.724
PRBC Transfusion	0 [0–0]	0 [0–0]	0 [0–0]	1.00

Abbreviations: BMI, body mass index; ASA, American Society of Anesthesiologists; OP, operation; PRBC, packed red blood cells.

**Table 2:** Return of bowel function, data presented as median and IQR.

Return of Bowel Function				
	All	OFA (*n* = 22)	Control (*n* = 22)	*p*-Value
First flatus (h)	44.0 [33.3–67.1]	43.0 [24.0–63.0]	50 [37.5–68.9]	0.219
First defecation (h)	83.5 [58.3–122.1]	62.5 [45.8–80.8]	118.5 [82.6–142.3]	<0.0001
Fluid balance on POD 2 (mL)	0.700 [−0.525–1.650]	0.500 [−0.600–1.600]	0.800 [−0.400–1.800]	0.544
Fluid balance on POD 3 (mL)	0.000 [−0.500–1.300]	0.600 [−0.500–2.500]	−0.100 [−0.625–0.750]	0.660

## Data Availability

Data are unavailable due to privacy or ethical restrictions.
